# Diurnal control of H3K27me1 deposition shapes expression of a subset of cell cycle and DNA damage response genes

**DOI:** 10.1111/tpj.17114

**Published:** 2024-11-01

**Authors:** Jorge Fung‐Uceda, María Sol Gómez, Laura Rodríguez‐Casillas, Anna González‐Gil, Crisanto Gutierrez

**Affiliations:** ^1^ Centro de Biologia Molecular Severo Ochoa, CSIC‐UAM Nicolas Cabrera 1, Cantoblanco 28049 Madrid Spain

**Keywords:** H3K27me1, ATXR5/6, chromatin, histone modification, gene expression, diurnal cycles, DNA repair, euchromatin, Arabidopsis

## Abstract

Rhythmic oscillation of biological processes helps organisms adapt their physiological responses to the most appropriate time of the day. Chromatin remodeling has been described as one of the molecular mechanisms controlling these oscillations. The importance of these changes in transcriptional activation as well as in the maintenance of heterochromatic regions has been widely demonstrated. However, little is still known on how diurnal changes can impact the global status of chromatin modifications and, hence, control gene expression. In plants, the repressive mark H3K27me1, deposited by ARABIDOPSIS TRITHORAX‐RELATED PROTEIN 5 and 6 (ATXR5 and 6) methyltransferases, is largely associated with transposable elements but also covers lowly expressed genes. Here we show that this histone modification is preferentially deposited during the night. In euchromatic regions, it is found along the bodies of DNA damage response genes (DDR), where it is needed for their proper expression. The absence of H3K27me1 translates into an enhanced expression of DDR genes that follows a rhythmic oscillation pattern. This evidences a link between chromatin modifications and their synchronization with the diurnal cycle in order to accurately modulate the activation of biological processes to the most appropriate time of the day.

## Significance Statement

We ask here how diurnal changes impact chromatin modifications and gene expression. We have found that H3K27me1 is preferentially deposited during the night along the bodies of cell cycle and DNA damage response genes. Our study highlights the relevance of the diurnal control of the chromatin landscape of genes involved in genome maintenance.

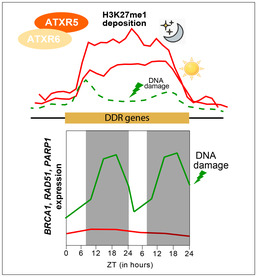

## INTRODUCTION

Organisms need to guarantee genome stability in order to allow proper development and to transmit accurately the genetic material to their progeny. This requires a tight control of faithful duplication during the cell cycle as well as of the chromatin landscapes that modulate gene expression (Takatsuka, Shibata, & Umeda, 2021). In the nucleus, DNA is wrapped around a family of well conserved proteins known as histones. Histone post‐translational modifications (PTMs) have proven to be essential not only in the activation/repression of genes but also in assuring chromatin stability (Jacob, Stroud, Leblanc, et al., 2010; Kouzarides, 2007).

In plants, histone modifications are essential in a plethora of biological processes (Foroozani, Holder, & Deal, 2022). An example of its importance is the response to changes in light–dark cycles, where a specific chromatin modification signature is needed for proper chromocenter architecture (Bourbousse et al., 2015; van Zanten, Tessadori, Peeters, & Fransz, 2012) or in the correlation with circadian clock gene expression along the day (Malapeira, Khaitova, & Mas, 2012; Maric & Mas, 2020; Seo & Mas, 2014). Synchronization with the 24‐h light–dark cycles confers organisms the capacity to adapt their biological processes to the most accurate time of the day (Gachon, Nagoshi, Brown, Ripperger, & Schibler, 2004; Green, Tingay, Wang, & Tobin, 2002), therefore increasing their fitness.

The ARABIDOPSIS TRITHORAX‐RELATED PROTEIN 5 (ATXR5) and ATXR6 encode for two unique plant histone methyl‐transferases that redundantly monomethylate the canonical histone H3 at lysine 27 (H3K27me1; (Jacob et al., 2009)). ATXR5 and ATXR6 were identified by their ability to interact with the DNA replication protein PCNA (Raynaud et al., 2006). Its absence has severe effects on genome stability, causing chromocenter decondensation, de‐repression of a subset of transposable elements (TEs), DNA re‐replication, and upregulation of DNA damage response (DDR) genes (Feng et al., 2017; Hale et al., 2016; Jacob et al., 2009; Jacob et al., 2010; Pontvianne et al., 2012; Potok et al., 2022). While H3K27me1 is extensively associated with the maintenance of silent heterochromatin, recent findings show that it may have a role in regulating the expression of lowly expressed genes in euchromatin, for example, the DDR genes, where this mark is present along their bodies (Potok et al., 2022). These genes play an important role in guaranteeing genome stability by ensuring DNA repair and integrity in somatic and germ cells (Kim, 2019).

Even though raising evidence suggests that there is a wide‐genome response of histone modifications to light–dark cycles in genes not related to light or circadian regulation, a direct address of this question is missing. A tempting hypothesis is that fast changes in chromatin will precede or maintain gene regulation in coordination with the 24‐h light–dark cycles to ensure that biological processes are active when needed during the diurnal cycle. Here we have studied the diurnal regulation of the repressive mark H3K27me1 and its role in the control of DDR gene expression as a novel regulatory layer dependent on its localization in euchromatic genomic regions.

## RESULTS

### The double mutant in the *ATXR5* and *ATXR6* genes displays a strong growth phenotype under specific photoperiodic conditions

The ATXR5 and ATXR6 histone methyltransferases are needed to prevent unscheduled expression of a subset of transposable elements (TEs) and the excess of DNA replication associated with heterochromatin (Feng et al., 2017; Jacob et al., 2010). In addition to the ATXR5/6‐mediated deposition of H3K27me1 in TEs, it is also present in euchromatic locations (Ma et al., 2018; Potok et al., 2022). It has been reported that the double mutant in both *ATXR5* and *ATXR6* genes (thereafter *atxr5/6*) displays abnormal growth with small leaves that impact overall plant size (Jacob et al., 2009). To investigate the impact of the H3K27me1 mark on plant growth, we first studied in detail leaf growth under different photoperiod conditions. Remarkably, the growth phenotype of the *atxr5/6* mutant was observed only in seedlings grown under short‐day (ShD; 8 h light, 16 h dark) conditions that led to a significant reduction in leaf area at both early (9 day‐old seedlings; Figure S1) and late (24 day‐old seedlings; Figure S1) growth times. On the contrary, no changes in leaf area were observed when seedlings were grown under long‐day conditions (16 h light, 8 h dark; Figure S1). Since the reduced size in *atxr5/6* leaves is observed throughout different stages of development, we conclude that the absence of ATXR5 and ATXR6 is needed for proper leaf development in days with longer dark periods. To evaluate the specific dependence of the *atxr5/6* leaf phenotype on the light regime, we determined root growth and found no significant difference in root length between *atxr5/6* and the wild type under short‐ and long‐day conditions (Figure S1). The leaf phenotype under short‐day conditions, which is not observed in roots, is consistent with (i) the different expression patterns of *ATXR5* and *ATXR6* (Raynaud et al., 2006 and our data) and (ii) the high levels of *ATXR6* expression in the *atxr5/6* mutant roots (Potok et al., 2022), which otherwise do not display upregulation of TEs and increased re‐replication (Vergara et al., 2023), typical phenotypes described in mature leaves of the *atxr5/6* mutant (Jacob et al., 2010). Therefore, our results support the conclusion that the reduced leaf size phenotype observed in *atxr5/6* in short days is due to an impaired interaction with the changing photoperiod.

### *ATXR5* gene expression and H3K27me1 levels oscillate diurnally

**Figure 1:**
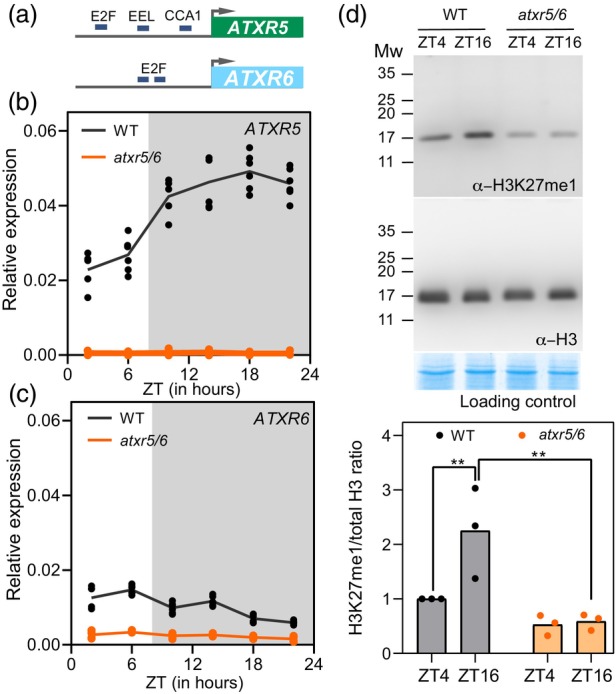
*ATXR5* and H3K27me1 oscillate diurnally. (a) Scheme of proximal regions of *ATXR5* and *ATXR6* genes showing the locations of E2F, EEL, and CCA1 binding sites. Diurnal time‐course of (b) *ATXR5* and (c) *ATXR6* expression in wild‐type (WT) and *atxr5/6* mutant plants. Relative expression was obtained by real‐time qPCR analyses. The original data points of each of the two biological replicates are shown together with the line joining the mean values. (d) Western blot analyses with anti‐H3K27me1 (upper panel) and anti‐H3 (lower panel) in wild type (WT) and *atxr5/6* at ZT4 and ZT16. Coomassie blue staining was used as loading control. Plants were grown under short‐day conditions for 14 DAS. The analysis of three biological replicates carried out is plotted, and a representative case of them is shown. Asterisks indicate statistically significant differences applying Two way ANOVA; ** *P* ≤ 0.01

We next examined the possible connection between ATXR5 and ATXR6 with the circadian clock, the endogenous mechanism in charge of translating day‐night transitions into rhythmic molecular and biological oscillations (Greenham & McClung, 2015). First, we carried out an in silico analysis to identify putative transcription factor binding sites in the upstream genomic regions of these two genes. We found that, in addition to E2F binding sites (TTssCG) in both *ATXR5* and *ATXR6* gene promoters, consistent with their cell cycle‐dependent expression (Raynaud et al., 2006), *ATXR5*, but not *ATXR6*, possessed two types of circadian clock DNA binding sites in its promoter region: a CCA1 binding site (CBS; AAAAATCT; (Wang et al., 1997)) and an evening element like (EEL; AATATCT; (Huang et al., 2012)) (Figure 1a).

To confirm the functional relevance of these circadian clock DNA binding sites on the rhythmic oscillations of *ATXR5* and *ATXR6* gene expression, we carried out a 24‐hour diurnal time‐course gene expression analysis under short‐day conditions. In the wild type, *ATXR5* displayed a clear diurnal oscillation with increasing levels peaking at night (ZT16; Figure 1b), whereas lower expression levels and no oscillation were observed for *ATXR6* (Figure 1c), fully consistent with data available in the diurnal project database (Figure S2) (Michael et al., 2008). As expected, both genes were downregulated and did not oscillate in the *atxr5/6* double mutant (Figure 1b,c). The diurnal oscillation observed only in *ATXR5* supported the hypothesis that both of these genes, besides sharing common functions, have functional specificities.

To assess the relevance of the oscillation in *ATXR5* gene expression, we asked whether H3K27me1 deposition also followed a diurnal oscillation. H3K27me1 levels were assessed in protein extracts of isolated nuclei at two time points, one in the middle of the day (ZT4) and another one in the middle of the night (ZT16), when the oscillation in the gene expression levels is better observed. H3K27me1 levels were lower in *atxr5/6* when compared to the wild type for both time points with no difference between them (Figure 1d). However, H3K27me1 in the wild type was higher at ZT16 than at ZT4, a result that matches the *ATXR5* diurnal gene expression (Figure 1d). We used as a control an antibody against total histone 3 (H3), which revealed no significantly different levels between genotypes or time points (Figure 1d).

Together, these results led us to conclude that *ATXR5* gene expression as well as de novo deposition of H3K27me1 display a diurnal regulatory layer, likely mediated by the circadian clock, that helps synchronize their activity in response to changes in the photoperiod.

### DDR genes upregulated in the *atxr5/6* mutant are enriched in H3K27me1

To get deeper knowledge on the impact of H3K27me1 during the day/night cycle, we first determined the H3K27me1 deposition pattern by chromatin‐immunoprecipitation followed by sequencing (ChIP‐Seq) and analyzed the H3K27me1 data normalized to total H3 across the genome. We found a significant reduction in the H3K27me1 signal in the *atxr5/6* mutant, in particular in the pericentromeric areas covered by TEs, as reported (Ma et al., 2018; Potok et al., 2022), both at midday and midnight (Figure S3). The signal across the chromosome arms, where most protein‐coding genes are located, is less clear due to the large bin size used in these metaplots. The analysis of H3K27me1 signal across the gene bodies revealed that many genes, both at ZT4 and ZT16, still maintained a significant H3K27me1 level (5024 and 4930 genes at ZT4 and ZT16, respectively; see Figure S3) in the *atxr5/6* mutant, likely due to its hypomorphic nature since the full loss of function of both genes is lethal (Potok et al., 2022).

**Figure 2:**
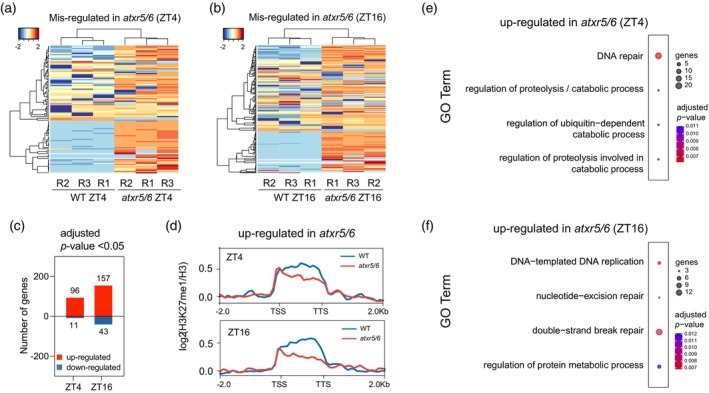
DDR genes are upregulated in *atxr5/6* and enriched in H3K27me1. (a, b) Clustered heatmaps of gene expression level in wild‐type (WT) and *atx5/6* double mutant. Significantly misregulated genes at (a) ZT4 and (b) ZT16 in *atxr5/*6 mutant compared to wild type were identified by RNA‐Seq. Genes were clustered by row and column according to the TPM values in individual biological replicates. Data were scaled by row and clustered following Euclidean distance. (c) Number of differentially expressed genes in wild‐type and *atxr5/6* seedlings at ZT4 and ZT16. (d) Profile of H3K27me1 ChIP‐Seq signal normalized to total H3 in wild‐type and *atxr5/6* seedlings along the bodies of upregulated genes in *atxr5/6* at ZT4 and ZT16, including 2 kb of flanking upstream and downstream regions. The read signal of ChIP‐Seq data was divided into bins of 25 bp. (e, f) Gene ontology (GO) term analysis for upregulated genes in *atxr5/6* at ZT4 (e; 96 genes) and ZT16 (f; 157 genes). The categories with a higher number of significant annotated genes are shown. Plants were grown under short‐day conditions for 14 DAS. Data shown were obtained from the analysis using three merged independent biological replicates (panels c, e, and f), except for the clustered heatmaps (panels a, b), where the results of each biological replicate are shown. Data shown on panel d were obtained from the analysis of two merged independent biological replicates.

To establish a link between H3K27me1 deposition and gene expression, we generated the transcriptomes of wild‐type and *atxr5/6* seedlings at ZT4 and ZT16 by RNA sequencing (RNA‐Seq). Since the focus of our study is on genes, we carried out the analysis after eliminating the sequences of TEs and TE‐genes (Figure S4). Independently of the time point analyzed, we identified more upregulated than downregulated protein‐coding genes in the *atxr5/6* double mutant (Figure 2a,b; Table S1). This suggests that H3K27me1 plays primarily a repressive role, in agreement with previous reports largely related to repression of TE expression (Ma et al., 2018; Potok et al., 2022). When looking at the day/night differences in detail, more genes were upregulated in the *atxr5/6* mutant at ZT16 (157 genes) than at ZT4 (96 genes) (Figure 2c; Table S1). These results correlated inversely with the higher *ATXR5* and H3K27me1 levels observed in the wild type at night (ZT16), suggesting again that H3K27me1 is a repressive mark acting especially during the night.

We then analyzed in detail the H3K27me1 signal across the bodies of *atxr5/6* misregulated genes. Compared to the wild type, our analysis revealed a clear decrease of H3K27me1 in the bodies of upregulated genes of *atxr5/6* at both ZT4 and ZT16 (Figure 2d). On the contrary, most of the downregulated genes do not show H3K27me1 deposition. These data, together with the RNA‐Seq data, strongly reinforce the repressive role of H3K27me1 on a subset of target genes, and inhibition is relieved in the *atxr5/6* mutant, leading to the activation of otherwise lowly expressed genes. This is consistent with the anticorrelation reported between H3K27me1 and gene expression (Jacob et al., 2010). To investigate the possible specificity of gene repression exerted by H3K27me1, we used a gene ontology analysis (GO) on the 96 and 157 upregulated genes in the *atxr5/6* mutant at ZT4 and ZT16, respectively (Figure 2e,f). A similar analysis of the downregulated gene is shown in Figure S5. Remarkably, most categories of upregulated genes at both time points were related to DNA replication, DNA repair, double‐strand break (DSB) repair, and response to radiation processes.

In spite of the high number of genes that contain H3K27me1 in the *atxr5/6* mutant, we were able to identify those with a complete loss of H3K27me1 signal in the mutant (56 genes at ZT4 and 103 genes at ZT16). The H3K27me1 deposition profile across these genes clearly showed that this mark was lost along the gene bodies (Figure S6), while a similar but less acute pattern was found in genes with a reduced level of H3K27me1 (see Figure 2d). Importantly, several genes involved in DNA repair and general DNA metabolism, such as *BRCA1*, *RAD51*, *PARP1*, *PARP2*, *SMC6b*, and *SMC2*, among others, are found in this group. The reason for some genes to respond differently to a reduction or loss of H3K27me1 is not presently known. Together, our transcriptomic and H3K27me1 ChIP‐Seq data revealed that H3K27me1 plays a repressive role on a subset of genes overrepresented in DDR processes. This repression seems to be higher at midnight (ZT16) than at midday (ZT4), pointing to the functional relevance of these genes, especially during the night.

### DDR genes, upregulated in *atxr5/6*, are diurnally oscillating

**Figure 3:**
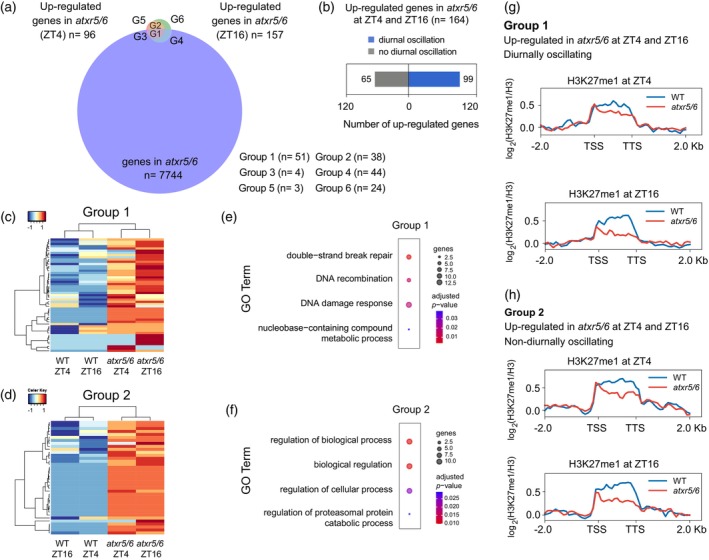
DDR genes are upregulated and diurnally oscillating in *atxr5/6*. (a) Venn diagram showing the overlap between upregulated genes at ZT4, at ZT6, and diurnally oscillating in *atxr5/6*. Groups 1 to 6 are indicated as G1–G6. (b) Number of upregulated genes also displaying diurnal oscillation in *atxr5/6*. (c, d) Clustered heatmap of gene expression level in wild type (WT) and *atxr5/6* for groups 1 (c) and 2 (d). Significantly misregulated genes at ZT4 and ZT16 in *atxr5/6* compared to wild type were identified by RNA‐Seq. Genes were clustered by row and column according to the mean TPM values of three biological replicates. Data were scaled by row and clustered following Euclidean distance. (e, f) GO term analysis of upregulated genes in *atxr5/6* of groups 1 (e, 51 genes) and 2 (f, 38 genes). The 4 categories with a higher number of significant annotated genes are shown. (g, h) Profile of H3K27me1 ChIP‐Seq signal normalized to total H3 in wild type (WT) and *atxr5/6* along the bodies of *atxr5/6* upregulated genes in groups 1 and 2, including 2 kb of flanking upstream and downstream regions. Reads of ChIP‐Seq data were divided into bins of 25 bp. Plants were grown under short‐day conditions for 14 DAS. Data shown were obtained from the analysis using the merging of two independent biological replicates.

To further define the H3K27me1‐mediated diurnal regulation of DDR and cell cycle genes, we compared the set of genes upregulated at ZT4 (96 genes) and ZT16 (157 genes) in *atxr5/6* with those diurnally oscillating in the same mutant (7744 genes; Figure 3a). We observed that ~60% of the upregulated genes in *atxr5/6* were found in those diurnally oscillating, while ~40% did not (Figure 3a,b). This analysis allowed us to classify the genes upregulated in the *atxr5/6* mutant into different groups. Here, we focused primarily on groups 1 (51 genes) and 2 (38 genes), which represented diurnally oscillating genes that are upregulated in *atxr5/6* (group 1), both at ZT4 and ZT16, and those displaying upregulation without diurnal oscillation at both ZTs (group 2; Figure 3c,d; Table S2). GO analysis revealed that most genes in group 1 were related to DDR, recombination, and genome maintenance, for example, *BRCA1*, *PARP1*, *RAD51*, and *GMI1*, while those in group 2 were enriched in various metabolic processes, for example, *ACC2*, *CYCB1;5, KRP6* (Figure 3e,f). These results suggested that even though both groups of genes are upregulated in the absence of ATXR5 and ATXR6, the diurnal layer of regulation is specific to DDR genes present in group 1. Genes of other groups, which were not related to the diurnal upregulation studied here, are shown in Figure S7 (see also Table S2).

We also examined the enrichment of H3K27me1 in the different groups and found that H3K27me1 is enriched in the body of genes of groups 1 and 2 in the wild type and reduced in *atxr5/6* (Figure 3g,h). This indicates that the presence of H3K27me1 seems to be a common repressive mark for all of them. In both cases, the apparent increase of the H3K27me1 signal observed at the TSS, consistent with previous reports (Jacob et al., 2010), is largely due to the decrease within the gene bodies, but the role of this pattern is unknown and should deserve a detailed analysis in the future. Other groups showed two different H3K27me1 profiles. For group 4, H3K27me1 levels were similar in wild type and *atxr5/6* (Figure S7), whereas for group 6, H3K27me1 levels were higher in the wild‐type than in the *atxr5/6* mutant (Figure S7). However, none of these genes were upregulated in *atxr5/6* during the day and night simultaneously with respect to the wild type (Figure S7), and the GO analysis did not reveal a clear representation of any functional group (Figure S7). We have not discussed groups 3 and 5 because the number of genes in these groups was too small. A likely possibility is that changes in the expression of genes in groups 3–6 are an indirect consequence of the mutations in the *ATXR5* and *ATXR6* genes. Therefore, our transcriptomic and ChIP‐Seq results support the conclusion that the H3K27me1 mark acts as a repressor of genes involved in a variety of biological processes, but, importantly, a subset of DDR genes are the only ones having a diurnal layer of regulation.

### Diurnal oscillation of DDR genes in *atxr5/6* correlates with changes in H3K27me1 levels

**Figure 4:**
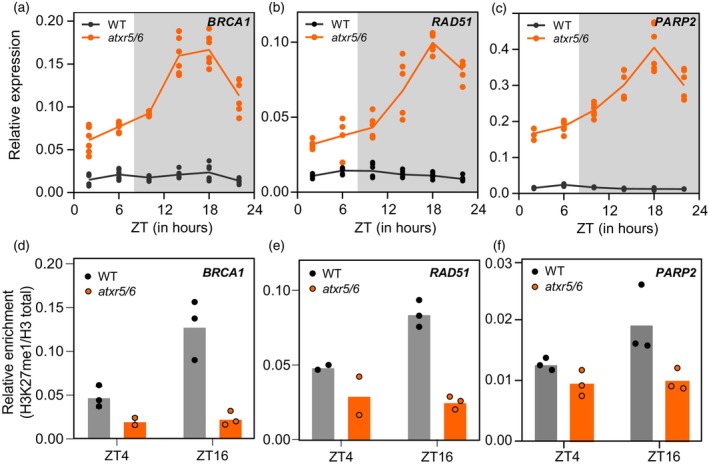
Diurnal oscillation of DDR genes in *atxr5/6* is correlated with H3K27me1 levels. Diurnal time‐course expression of *BRCA1* (a), *RAD51* (b), and *PARP2* (c) in wild‐type (WT) and *atxr5/6* plants. Relative expression was obtained by real‐time qPCR analyses. Individual data points from two biological replicates are shown together with the line joining the mean values. (d–f) ChIP‐qPCR assays in wild‐type (WT) and *atxr5/6* plants at ZT4 and ZT16 using an anti‐H3K27me1 antibody. H3K27me1 ChIP enrichment was calculated relative to total H3 values. Bars represent mean values of three (data points) technical replicates of one of the two biological replicates performed.

DDR has been well studied in response to genotoxic agents or environmental stresses (Hu, Cools, & De Veylder, 2016; Nisa, Huang, Benhamed, & Raynaud, 2019). To decipher the mechanism of the diurnal gating of DDR, we measured the expression level of a subset of DDR genes upregulated at ZT4 and ZT16 and diurnally oscillating in *atxr5/6*, based on our RNA‐seq data. To get a direct insight into this regulatory mechanism, we carried out 24 ‐h time‐course measurements of the mRNA levels of selected genes involved in the DDR pathway. Genes involved in DSB repair, such as *BRCA1* (Figure 4a), *RAD51* (Figure 4b), and *PARP2* (Figure 4c), were activated and diurnally oscillating in the *atxr5/6* mutant. Their expression level increased toward and during the night, with a peak at ~ZT16‐ZT18 (Figure 4a–c). However, expression of other DDR genes not involved in DSB, such as *TEJ* and *KU80* (Figure S8a,b), did not apparently differ from the wild type and/or displayed a different pattern of oscillation.

Mutations in the *ATXR5* and *ATXR6* genes and the subsequent reduction in H3K27me1 levels over target genes seemed to be sufficient for the diurnal oscillation of DSB repair genes. The presence of circadian clock binding sites at the *ATXR5* promoter region further pointed to this mechanism to be, at least partially, responsible for their oscillation. However, to rule out that the rhythmicity in DDR gene expression was an indirect cause of an impaired function of the circadian clock in the *atxr5/6* mutant caused by the genome‐wide lowering of H3K27me1 levels, we performed 24 ‐h time‐course analysis of two core clock genes, *CCA1* and *TOC1*. We found that these genes did not display any oscillatory or amplitude difference between the wild‐type and the *atxr5/6* mutant (Figure S8c,d). This reinforces our conclusion that the absence of H3K27me1 is directly responsible for the activation and diurnal oscillation of DDR genes. ChIP‐qPCR assays further supported this conclusion since a clear decrease in the H3K27me1 enrichment at DDR genes, such as *BRCA1* (Figure 4d), *RAD51* (Figure 4e), and *PARP2* (Figure 4f), was detected in *atxr5/6*, without an increase at night (ZT16) as it occurs in wild‐type plants (Figure 4d–f). The latter correlated inversely with the peak of DDR gene expression in *atxr5/6*, indicating a repressive function of this mark, especially during the night.

### DDR genes oscillate diurnally in the wild type after exposure to bleomycin

**Figure 5:**
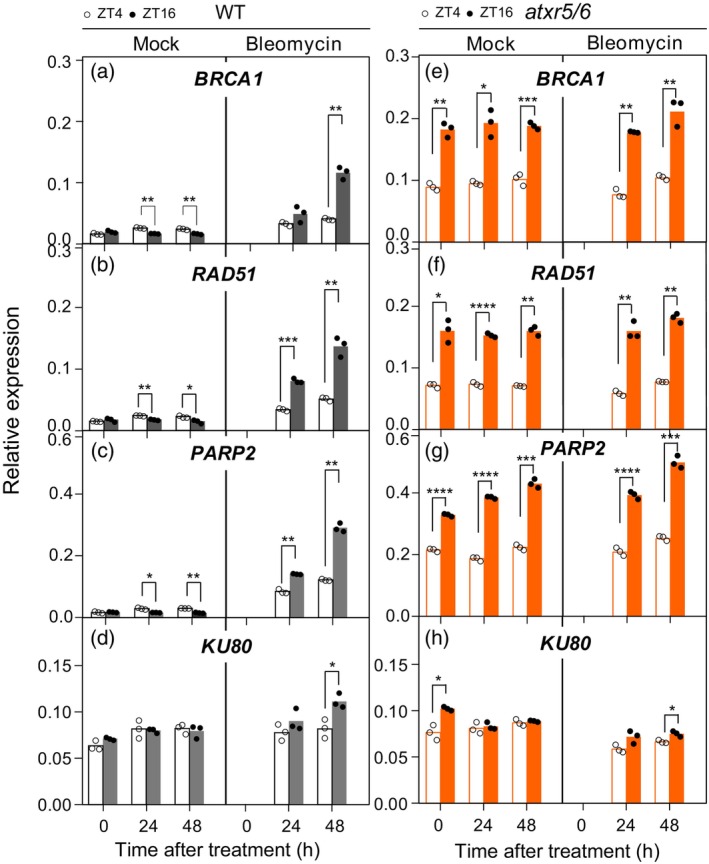
DDR genes oscillate diurnally in the wild type (WT) after exposure to genotoxic agents. (a, e) *BRCA1*, (b, f) *RAD51*, (c, g) *PARP2,* and (d, h) *KU80* expression in (a–d) wild‐type (WT) and (e–h) *atxr5/*6 plants at ZT4 and ZT16 after bleomycin or mock treatments. Plants were grown under a short‐day regime for 14 DAS. Relative expression was obtained by real‐time qPCR analyses. Bars represent mean values of three (data points) technical replicates of one of the two biological replicates performed. Asterisks indicate statistically significant differences applying a Welch's test; *P ≤ 0.05, **P ≤ 0.01, ***P ≤ 0.001, ****P ≤ 0.0001.

To test whether the diurnal oscillation of DSB repair genes in *atxr5/6* was mimicking a true DNA damage response, we treated wild‐type plants with bleomycin, a DSB‐producing agent, for prolonged periods (up to 2 days) and followed the expression of three DSB genes (*BRCA1*, *RAD51*, and *PARP2*) and one not involved directly in the DSB response (*KU80*; (Takatsuka et al., 2021)), at two time points (ZT4 and ZT16). As expected, the three DSB repair genes analyzed showed an oscillatory pattern of expression in the wild type with higher levels at ZT16 (Figure 5a–c). In contrast, no differences were observed between mock and bleomycin‐treated plants in the *atxr5/6* mutant, where they continued to display their oscillatory pattern of expression with higher levels during the night (Figure 5e–g). Neither induction nor oscillation was seen for *KU80* after treatment with bleomycin in both wild‐type and *atxr5/6* mutants (Figure 5d,h), demonstrating the specificity of the response to DSB repair genes. This response does not come from a misregulation of the circadian clock function, as shown by the maintenance of the amplitude and oscillation of *CCA1* and *TOC1* in the wild type after treatment with bleomycin (Figure S9). Our results indicated that the diurnal oscillation of DSB repair genes in *atxr5/6* is mimicking the response of the wild type to exposure to low doses of bleomycin.

## DISCUSSION

Chromatin modifications, for example, post‐translational modifications of histones, are at the core of genome organization and dynamics, and they have a key impact on gene regulation (Pikaard & Mittelsten Scheid, 2014). Thus, histone PTMs are associated with either active gene expression (H3K4me3, H3K36me3, among others) or gene silencing (H3K9me2, H3K27me1 in plants) in a myriad of biological processes. Interestingly, specific chromatin signatures correlate with the fine‐tuning of gene expression in global and tissue‐specific scenarios. Ultimately, the combination of different PTMs can indicate the active or repressive state of gene expression (Sequeira‐Mendes et al., 2014).

Diurnal oscillation of biological processes is crucial for all organisms to adapt their physiology to the day/night changes. This adaptation, which is of primary relevance in plants, depends on strictly and finely regulated control of gene expression of a cohort of genes, post‐translational modifications, and the deposition of specific chromatin marks on circadian clock gene loci (Nohales & Kay, 2016). Here we have shown a link between diurnal regulation of cell cycle and DNA repair genes with their silencing mediated by changes in H3K27me1 in *Arabidopsis*. Our findings highlight the importance of the cross‐talk between different layers of regulation in the fine control of gene expression as a response to the changing environment.

The *ATXR5* (ARABIDOPSIS TRITHORAX‐RELATED5) and *ATXR6* genes encode for histone methyltransferases, unique to plants, that produce H3K27me1 (Jacob et al., 2009). This mark is crucial for chromocenter condensation and repression of subsets of transposable elements (Jacob et al., 2009; Jacob et al., 2010; Potok et al., 2022; Vergara et al., 2023). In addition, it is necessary to prevent extensive DNA re‐replication of heterochromatic regions (Feng et al., 2017; Jacob et al., 2010). Although H3K27me1 is largely associated with heterochromatin silencing, its absence also leads to increased expression of DNA damage response (DDR) genes (Hale et al., 2016; Jacob et al., 2009; Ma et al., 2018; Stroud et al., 2012). This could occur as a consequence of multiple chromatin defects and/or as a specific association of H3K27me1 with gene expression control.

In addition to typically colocalizing with heterochromatin, H3K27me1 is also present in lowly expressed or silenced protein‐coding genes (Jacob et al., 2010; Potok et al., 2022). Here we have focused on the dynamics of H3K27me1 in these genes. First, we confirmed that *ATXR5* gene expression is diurnally regulated, with a peak of expression in the middle of the night. This is consistent with the presence of CCA1 and EEL binding sites in its promoter, which are not present in the *ATXR6* gene. The diurnal regulation leading to high *ATXR5* expression at night also leads to higher levels of H3K27me1. It is noteworthy to indicate that the diurnal oscillation observed and the growth defects displayed under specific photoperiod conditions point to a close interaction with changes in the environment. Changes in the photoperiod follow annual seasonal changes in nature, and plants sense them by pace adaptation of their circadian clock (Schultz & Kay, 2003; Song, Shim, Kinmonth‐Schultz, & Imaizumi, 2015).

To get deeper knowledge of the impact of H3K27me1 deposition on protein‐coding genes when plants grow under short‐day conditions, we determined its distribution pattern at ZT4 and ZT16 by ChIP‐Seq. Due to the hypomorphic nature of mutations in the *atxr5/6* plants used in this work, we found that many genes still show a H3K27me1 signal, although its deposition shows a notable decrease mainly at midnight when compared to wild‐type plants.

To determine the impact of H3K27me1 on global gene expression, we generated RNA‐Seq data of both wild‐type and *atxr5/6* mutant plants at midday (ZT4) and midnight (ZT16). This analysis allowed us to identify genes of the DDR as a subset primarily affected in their expression at midnight in the *atxr5/6* mutant, consistent with a reduction in their H3K27me1 levels. This reduction, which appears to affect gene expression, occurs primarily across the gene bodies, affecting the overall profile and producing an apparent signal at the TSS. Since H3K27me1 is a substrate of PRC2 to generate H3K27me2/3, changes in the H3K27me1 levels might be relevant for PRC2 target genes. We can discard this possibility since a very small percentage of reported PRC2 target genes are upregulated in the *atxr5/6* mutant (1.25%–2.9% depending on the reported lists of PRC2 targets) (Antunez‐Sanchez et al., 2020; Lafos et al., 2011; Roudier et al., 2011). Furthermore, DDR and DNA metabolism‐related genes are not present within this small group of genes. Levels of H3K27me1 might also be affected by the activity of H3K27me3 demethylases such as REF6 and ELF6. However, none of the genes upregulated in the *atxr5/6* mutant at ZT4 and ZT16 that overlap with reported REF6/ELF6 targets (Antunez‐Sanchez et al., 2020) are associated with DDR.

Among all the diurnally oscillating and H3K27me1‐regulated DDR genes, we identified several of them required for double‐strand break (DSB) repair and homologous recombination (HR), such as *BRCA1*, *RAD51,* and *PARP2*. However, genes acting in non‐homologous end‐joining (NHEJ), such as KU80, were not affected. This confirms that H3K27me1 dynamics seems to specifically affect the subset of DDR genes involved in HR. Notably, the increased expression of the HR genes in the *atxr5/6* mutant, but not of KU80, correlates with decreasing levels of H3K27me1 more strongly during the night (ZT16). The increased expression levels of DSB repair genes, such as RAD51, in the *atxr5/6* also lead to geminivirus resistance in plants grown under short‐day conditions (Wang et al., 2023).

Our findings indicate that the H3K27me1 dynamics are important to control DDR gene expression in a diurnally oscillating manner. The specificity by which only DDR genes involved in HR were repressed by H3K27me1 and diurnally oscillating in mutants impaired in its deposition made us explore the possibility of a crosstalk between gene expression control by histone modification and circadian regulation in order to respond to external stimuli causing DNA damage. Therefore, we also sought to investigate the expression response of the subset of genes after a DNA‐damaging treatment of wild‐type plants. We found that the expression level of HR genes (*BRCA1*, *RAD51*, and *PARP2*) in wild type after treatment with bleomycin mimicked the pattern observed in the *atxr5/6* mutant. This supports the conclusion that DDR activation in the *atxr5/6* mutant is the consequence of deregulation of H3K27me1. It is also worth noting that the diurnal oscillation of DSB repair genes in the mutant is not the result of a side effect on the circadian clock derived from a reduction in H3K27me1 levels, since both *CCA1* and *TOC1* genes maintained their expression pattern, amplitude, and oscillation after treatment of wild‐type plants with bleomycin. Moreover, these results indicated that H3K27me1 may be acting as a “repressive switch” in order to prevent the unnecessary expression of HR DDR genes and that upon DNA damage agents, it is lifted in order to allow their proper expression.

Our results expand the knowledge of how deposition of a silencing mark is linked to the diurnal control of gene expression. In particular, we provide evidence of the relevance of diurnal regulation of H3K27me1 deposition on the expression of a subset of genes that are enriched in cell cycle and DNA repair functions. We have also provided insights on the complexity of the plant response to the changing environment by showing how a chromatin mark can follow diurnal changes in different parts of the genome in order to influence the transcription status of a role‐specific group of genes. It is worth noting that the photoperiod‐specific growth defects in a mutant impaired in H3K27me1 deposition point to a close interaction between histone marks and circadian regulation in order to accurately prepare the plant's response to external stimuli (Dornbusch, Michaud, Xenarios, & Fankhauser, 2014; Walter, Silk, & Schurr, 2009).

However, some questions still remain to be answered, for example and most importantly, what is the functional relevance of DDR gene oscillation in the *atxr5/6* mutant. We think this may be because of the coordination between cellular processes and stress response in order to maintain genome integrity during replication, when DDR gene functions may be needed (Bonnot, Blair, Cordingley, & Nagel, 2021; Seo & Mas, 2015). The idea of DNA repair and cell cycle genes being restricted to a specific window of the day to avoid DNA damage caused by sunlight has been long proposed with little evidence shedding any light (Goto & Johnson, 1995; Serrano et al., 2009). Our results showing diurnal oscillation of DDR genes in the wild type under genotoxicity indicate that the interplay of these processes may be more complex than previously suspected and that many other agents may be also involved. Given the importance of a correct function of DNA repair pathways for genome maintenance and stability (Hu et al., 2016; Takatsuka et al., 2021), the H3K27me1‐mediated diurnal control of DDR gene expression shed light on an additional layer of regulation allowing the plant to fine‐tune gene expression to the most appropriate time of the day.

Finally, another important question derived from our findings is how the higher levels of H3K27me1 found during the night are reduced during the day. This is not easy to answer, although there are several possibilities: (i) the presence of a so far unidentified demethylase; (ii) the incorporation of newly synthesized histones during DNA replication could provide a way to introduce in chromatin histones lacking the H3K27me1 mark; and (iii) likewise, active transcription could also be a mechanism to exchange histones. Perhaps the latter is more likely to occur, although a deeper and future study could shed light on the mechanism(s) operating.

## EXPERIMENTAL PROCEDURES

### Growth conditions and plant material

*Arabidopsis thaliana* seedlings were grown in Murashige and Skoog (MS) with 0.8% agar medium. Seedlings were synchronized in short days (8 h light – 16 h dark) or long days (16 h light – 8 h dark), as specified in each experiment, at 22°C. Wild type (Col‐0) and *atxr5/6* (Jacob et al., 2009) have been used here.

### Leaf size measurements

For leaf size measurements, at least 30 #1/#2 leaves of seedlings grown under short days or long days were harvested at the specified time points. Leaves were incubated in methanol over night for chlorophyll removal and then stored in lactic acid before microscopy analysis. The leaf blade area of the first pair of true leaves was measured using a wide‐field microscope (Zeiss) and quantified using the ImageJ software. Each experiment was repeated at least twice using a similar sample size.

### Root length measurements

Col‐0 and *atxr5/6 Arabidopsis thaliana* seeds were sown on MSS plates containing 1% agar and grown under short days or long days conditions at 22°C. Root length was measured daily over 10 days. The experiment was repeated at least twice using a similar sample size.

### Real‐time qPCR analysis

For expression analysis, 14‐day‐old seedlings were harvested at ZT4 and ZT16 or every 4 h during a 24‐h cycle for diurnal gene expression analysis. RNA was isolated using Maxwell® RSC Plant RNA Kit (Promega). Single‐strand cDNA was synthesized using iScript™ Reverse Transcription Supermix for RT‐qPCR (BioRad) following manufacturer instructions. For quantitative real‐time gene expression (qPCR), cDNA was diluted 10 times in nuclease‐free water and performed using iTaq Universal SYBR Green Supermix (BioRad) in a 384‐well CFX Opus3 Real‐Time PCR System (BioRad). The mean of IPP2 expression was used as a control. The crossing point (Cp) calculation was used for quantification using the absolute quantification analysis by the 2nd derivative maximum method. Each sample was run in technical triplicates, and each experiment was repeated twice. Primers used in these experiments are listed in Table S3.

### Nuclei isolation and western blot analysis

Nuclear protein extracts for Western blot were obtained from seedlings harvested at the indicated time points. For each sample, 14‐day‐old seedlings were snap‐frozen in liquid nitrogen and grounded using a mortar and a pestle. Samples were resuspended in rotation for 30 min with HONDA buffer (0.44 M Sucrose, 1.25% Ficoll, 2.5% Dextran T40, 20 mM HEPES KOH pH 7.4, 10 mM MgCl2, and 0.5% Triton X‐100) supplemented with 1X protease inhibitor cocktail (Sigma), 1 mM PMSF, and 100 μM MG132 (Calbiochem). Isolated nuclei were lysed in nuclei lysis buffer (50 mM Tris–HCl pH 8 and 10 mM EDTA) supplemented with 1X protease inhibitor cocktail (Sigma), 1 mM PMSF, and 100 μM MG132, followed by the addition of SDS to a final concentration of 2%. Nuclear protein extracts were quantified using the Pierce® BCA protein assay kit (ThermoScientific). All procedures were performed at 4°C.

Nuclear proteins were separated in a 15% Tris‐glycine protein gel and transferred to a 0.2 μM PVDF membrane (Amersham) using the Trans‐Blot Turbo Transfer System (BioRad). For H3K27me1 detection, 100 μg of total nuclear protein extract was loaded and 25 μg for H3. For immunoblot, anti‐H3K27me1 (07448 Millipore) and anti‐H3 (ab1791 Abcam) at a 1:1000 dilution in 5% milk were used. Secondary anti‐rabbit (GE NA9340V) was used at a 1:10000 dilution. Membranes were revealed with chemiluminescent HRP substrate (Millipore) in an AmershamTM Imager 680 (Amersham). The experiment was repeated three times.

### RNA‐seq library preparation, sequencing and analysis

Stranded RNA‐Seq libraries were generated from RNA isolated with miRNeasy Mini Kit® (Qiagen) and ribosome‐depleted by Ribo‐Zero Plant using the TruSeq Stranded Total RNA kit (Illumina) following the manufacturer's instructions. Three independent biological replicates were performed for each RNA‐Seq sample. All libraries were sequenced on the Illumina NovaSeq 6000 platform with double‐end 150 bp read length with a depth of 40 M reads.

RNA‐Seq raw reads were trimmed with Trimmomatic (v 0.39; (Bolger, Lohse, & Usadel, 2014)) to remove adapters and small sequences. Quality control of the raw (151 bp, paired‐end) and trimmed reads was assessed with FastQC (v.0.11.9; (Andrews, 2010)). Trimmed reads were aligned to the *A. thaliana* reference genome (TAIR 10, https://www.arabidopsis.org) with HISAT2 (v 2.1.0, −k 10 parameter; (Kim, Paggi, Park, Bennett, & Salzberg, 2019)). Unmapped reads and secondary alignments were filtered out with SAMtools (v1.9; (Danecek et al., 2021)). Gene abundances (TPMs and FPKMs) were counted by StringTie (v 2.2.1; (Shumate, Wong, Pertea, & Pertea, 2022)) using the Araport 11 GFF annotation file (https://www.arabidopsis.org). Differential gene expression (DGE) analysis was performed using DESeq2 (v 1.24.0; (Love, Huber, & Anders, 2014)). Genes with adjusted *P*‐value <0.05 were considered as statistically differentially expressed. Heatmaps representations were generated with R (version 4.1.2). Matplotlib (v 3.5.1; (Hunter, 2007)) was used to generate Venn diagrams. Gene ontology (GO) enrichment analysis was conducted using the topGO package (v 2.46.0; (Alexa & Rahnenfuhrer, 2023)). Heatmaps and barplots were generated with the R package ggplot2 (v 3.4.0; (Wickham, 2016)). All analyses were performed using the merge of all replicates.

### ChIP‐seq library preparation, sequencing and analysis

DNA for ChIP‐Seq was obtained after immunoprecipitation with anti‐H3K27me1 (07448 Millipore) and anti‐H3 (ab1791 Abcam). Libraries were generated using the Ovation® Ultralow V2 DNA‐Seq Library Preparation Kit (Tecan). Manufacturer instructions were carefully followed. Total ChIPed DNA was resuspended in 10 μL of nuclease‐free water and quantified by Qubit HS dsDNA assay (ThermoScientific) before the amplification step. 7–16 amplification cycles were used after taking into consideration ChIPed DNA concentration. Library quality was assessed by DNA BioAnalyzer (Agilent). One gram of seedlings was used as starting material. Two independent biological replicates were performed for each ChIP‐Seq sample. All libraries were sequenced on the Illumina NovaSeq 6000 platform with double‐end 150 bp read length with a depth of 40 M reads. All analyses were performed using the merge of all replicates.

ChIP‐Seq raw reads were trimmed with Trimmomatic (v 0.39; (Bolger et al., 2014)) to remove adapters and small sequences. FastQC (v 0.11.9; (Andrews, 2010)) was used to assess the quality control of the reads. Trimmed reads were then aligned to the *A. thaliana* reference genome (TAIR10, https://www.arabidopsis.org/) using Bowtie2 (v 2.3.4.3; (Langmead & Salzberg, 2012)) with default parameters. Duplicate reads were removed with Picard MarkDuplicates (v 2.7.1; https://broadinstitute.github.io/picard/). Unmapped reads and unwanted regions (transposons, miRNA) were removed with SAMtools (v 1.9; (Danecek et al., 2021)). Narrow peaks were called with MACS2 (v 2.2.6, additional parameters ‐p 0.05 ‐s 150; (Zhang et al., 2008)). DeepTools bamCompare (v 3.5.1; (Ramírez et al., 2016)) was used to normalize reads with CPM and against H3. Read pileup plots were generated with deepTools.

### Chromatin immunoprecipitation and qPCR analysis

Plants were grown under short day conditions and harvested at the specified time points. Chromatin immunoprecipitation (ChIP) was performed as described (Desvoyes, Vergara, Sequeira‐Mendes, Madeira, & Gutierrez, 2018). Briefly, samples were fixed in a 1% formaldehyde solution under vacuum (16% Formaldehyde (w/v), Methanol‐free, Thermo Fisher) for a total of 12 min. Soluble chromatin was incubated overnight at 4°C with anti‐H3K27me1 (07448 Millipore) and anti‐H3 (ab1791 Abcam) for the detection of these two proteins. Conjugates were immunoprecipitated for 4 h at 4°C with protein G Dynabeads™ (Thermo Fisher). ChIPs were quantified by qPCR using iTaq Universal SYBR Green Supermix (BioRad) in a 384‐well CFX Opus3 Real‐Time PCR System (BioRad). Crossing point (Cp) calculation was used for quantification using the absolute quantification analysis by the 2nd Derivative maximum method. Each sample was run in technical triplicates. Primers used for specific gene sequences can be found in Table S3. Each experiment was repeated at least twice.

### Bleomycin treatments

Twelve‐day‐old seedlings were grown in MS medium and supplemented with 1 μM bleomycin (Abcam). For q‐PCR analysis, seedlings were collected at 0, 24, and 48 h after treatment at ZT4 and ZT16. Immediately after, samples were snap‐frozen on liquid nitrogen for further processing. Plants treated with sterile water were used as mock controls. The experiment was repeated twice.

## Author Contributions

JF‐U and CG conceived the study. JF‐U and SG carried out the experiments with the participation of AG‐G. LR‐C and SG carried out the bioinformatic analysis. CG wrote the manuscript together with all authors.

## Supporting information


**Figure S1.** *atxr5/6* mutants display growth phenotype only under specific photoperiodic conditions.
**Figure S2.** Diurnal expression of *ATXR5* and *ATXR6* genes under short‐day growth conditions.
**Figure S3.** H3K27me1 profile along the Arabidopsis chromosomes and gene bodies.
**Figure S4.** Volcano plots of RNA‐seq experiments.
**Figure S5.** GO of downregulated genes.
**Figure S6.** H3K27me1 profile of upregulated genes in *atxr5/6* with detectable signal in wild type (WT) along the gene bodies.
**Figure S7.** Heatmaps, gene ontology analysis and H3K27me1 profile of genes in groups 4 and 6, described in Figure 3.
**Figure S8.** Diurnal time‐course expression of *TEJ* (A), *KU80* (B), *CCA1* (C) and *TOC1* (D) in wild‐type and *atxr5/6* plants along the day.
**Figure S9.** Diurnal oscillation of *CCA1* and *TOC1* genes after exposure to genotoxic agents.

**Table S1.** Up‐ and downregulated genes in the *atxr5/6* mutant, compared to wild type, at ZT4 and ZT16, as described in Figure 2.

**Table S2.** Genes included in groups 1–6, as described in Figure 3.

**Table S3.** List of primers used in this study.

## Data Availability

DNA sequencing datasets are available under accessions GSE261764 (RNA‐seq) and GSE261905 (ChIP‐seq).
