# Supplementary material for: Diurnal control of H3K27me1 deposition shapes expression of a subset of cell cycle and DNA damage response genes
Source: Plant J. 2024 Nov 1;120(6):2325–36. doi: 10.1111/tpj.17114 (PMC11658180; doi:10.1111/tpj.17114)
Supplement: Supplementary file 1 — Figure S1. atxr5/6 mutants display growth phenotype only under specific photoperiodic conditions. Figure S2. Diurnal expression of ATXR5 and ATXR6 genes under short‐day growth conditions. Figure S3. H3K27me1 profile along the Arabidopsis chromosomes and gene bodies. Figure S4. Volcano plots of RNA‐seq experiments. Figure S5. GO of downregulated genes. Figure S6. H3K27me1 profile of upregulated genes in atxr5/6 with detectable signal in wild type (WT) along the gene bodies. Figure S7. Heatmaps, gene ontology analysis and H3K27me1 profile of genes in groups 4 and 6, described in Figure 3. Figure S8. Diurnal time‐course expression of TEJ (A), KU80 (B), CCA1 (C) and TOC1 (D) in wild‐type and atxr5/6 plants along the day. Figure S9. Diurnal oscillation of CCA1 and TOC1 genes after exposure to genotoxic agents. [file TPJ-120-2325-s002.pdf]

Main Figures

Diurnal control of H3K27me1 deposition shapes expression of a subset of cell cycle and DNA damage response genes

Jorge Fung-Uceda[+], María Sol Gómez[+], Laura Rodríguez-Casillas, Anna González-Gil, Crisanto Gutierrez[*]

Centro de Biologia Molecular Severo Ochoa, CSIC-UAM, Nicolas Cabrera 1, Cantoblanco, 28049 Madrid, Spain

[+] These are co-first authors

* Corresponding autor: Crisanto Gutierrez
Email: cgutierrez@cbm.csic.es
Telephone: +34 911964638

Keywords: H3K27me1, ATXR5/6, chromatin, histone modification, gene expression, diurnal cycles, DNA repair, euchromatin, Arabidopsis

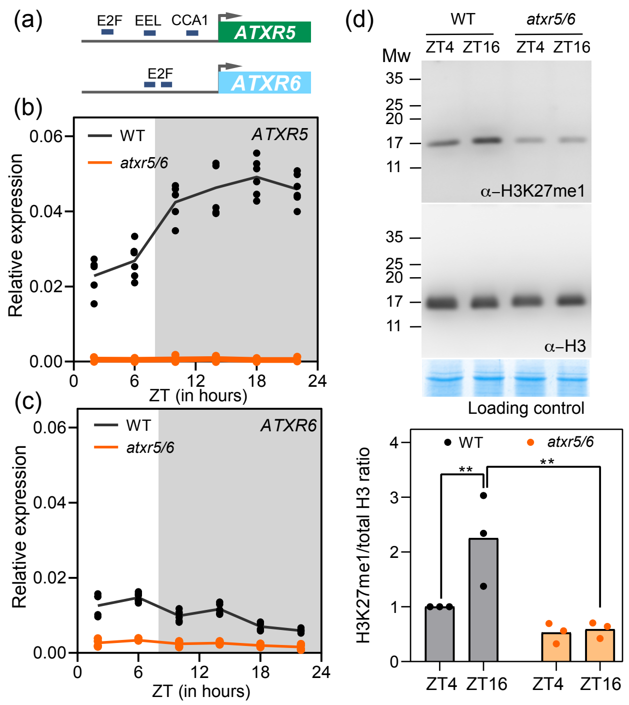

(a)
E2F
EEL
CCA1
ATXR5
E2F
ATXR6
(b)
WT
atxr5/6
ATXR5
Relative expression
ZT (in hours)
(c)
WT
atxr5/6
ATXR6
Relative expression
ZT (in hours)
(d)
WT
atxr5/6
ZT4 ZT16 ZT4 ZT16
Mw
α–H3K27me1
α–H3
Loading control
H3K27me1/total H3 ratio

Figure 1

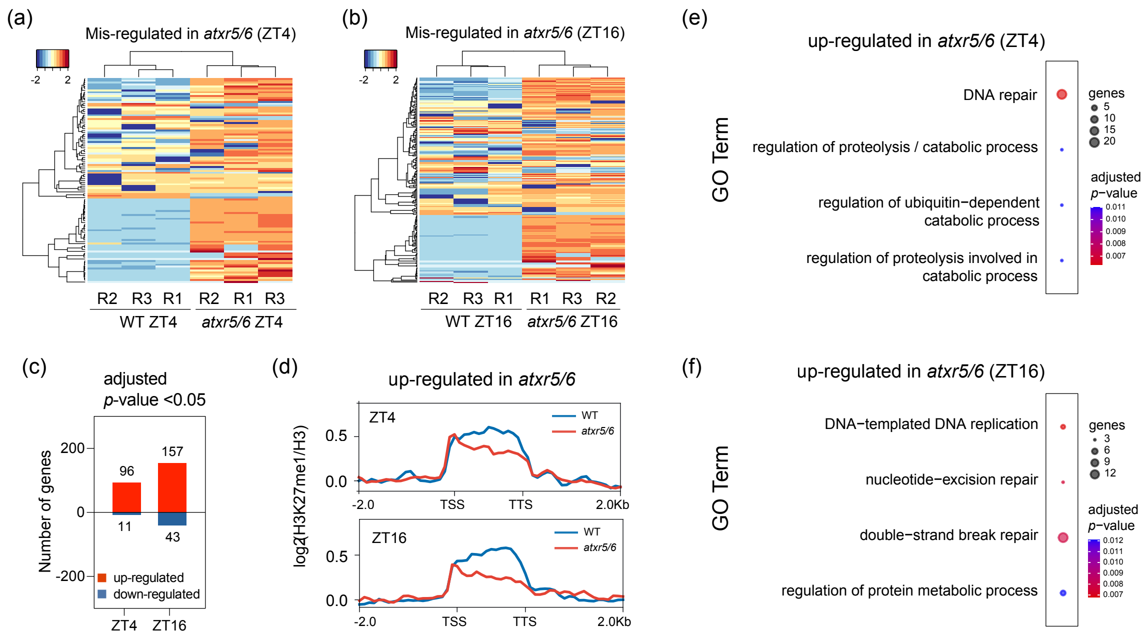

(a)
Mis-regulated in atxr5/6 (ZT4)
-2 2
R2 R3 R1 R2 R1 R3
WT ZT4 atxr5/6 ZT4
(b)
Mis-regulated in atxr5/6 (ZT16)
-2 2
R2 R3 R1 R1 R3 R2
WT ZT16 atxr5/6 ZT16
(c)
adjusted p-value <0.05
Number of genes
200
0
-200
96
157
11
43
up-regulated
down-regulated
ZT4 ZT16
(d)
up-regulated in atxr5/6
log2(H3K27me1/H3)
ZT4
WT
atxr5/6
0.5
0.0
-2.0 TSS TTS 2.0Kb
ZT16
WT
atxr5/6
0.5
0.0
-2.0 TSS TTS 2.0Kb
(e)
up-regulated in atxr5/6 (ZT4)
GO Term
DNA repair
regulation of proteolysis / catabolic process
regulation of ubiquitin−dependent catabolic process
regulation of proteolysis involved in catabolic process
genes
5
10
15
20
adjusted p−value
0.011
0.010
0.009
0.008
0.007
(f)
up-regulated in atxr5/6 (ZT16)
GO Term
DNA−templated DNA replication
nucleotide−excision repair
double−strand break repair
regulation of protein metabolic process
genes
3
6
9
12
adjusted p−value
0.012
0.011
0.010
0.009
0.008
0.007

Figure 2

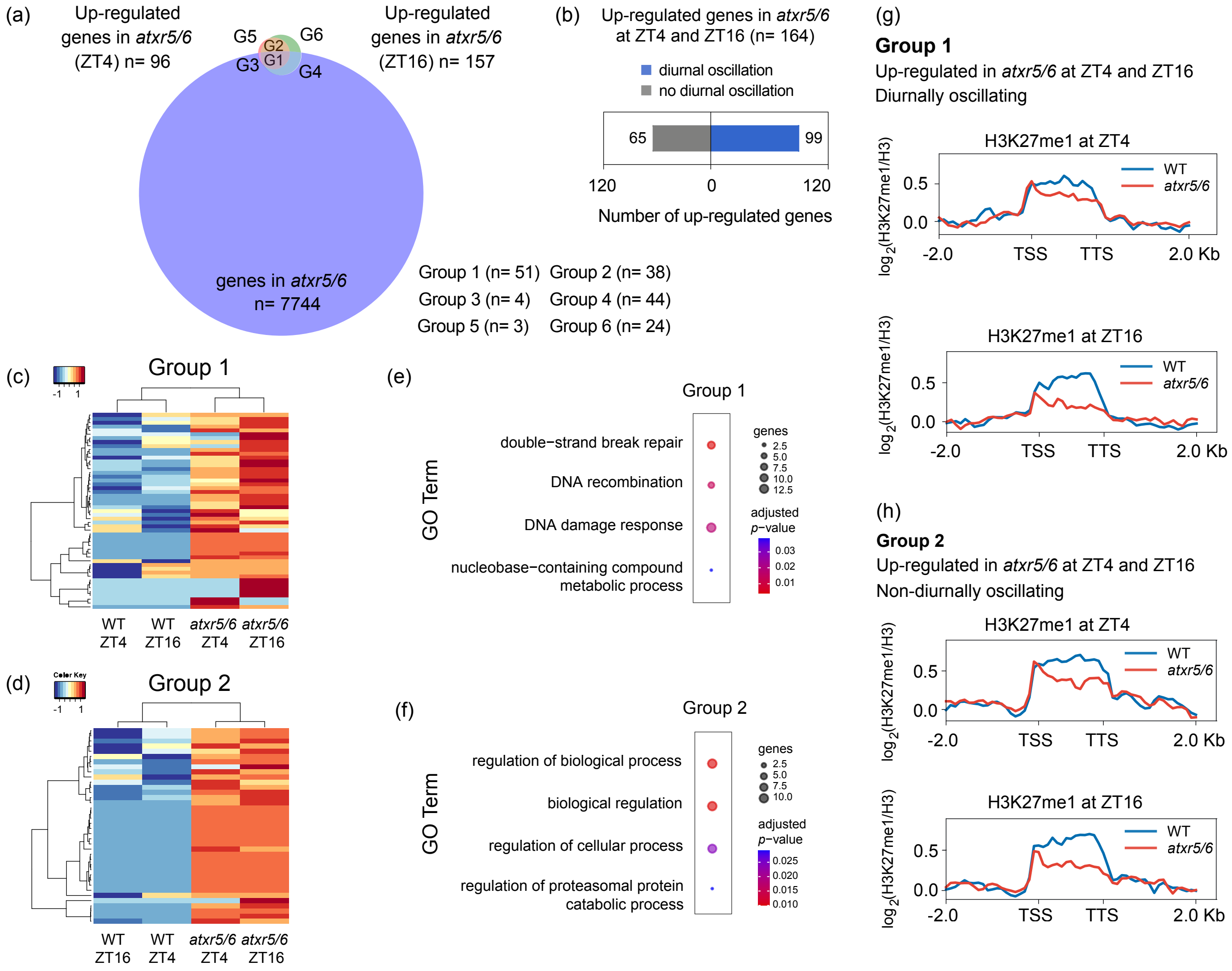
(a)
Up-regulated genes in atxr5/6 (ZT4) n= 96
Up-regulated genes in atxr5/6 (ZT16) n= 157
G5 G2 G6 G3 G1 G4
genes in atxr5/6 n= 7744
Group 1 (n= 51) Group 2 (n= 38)
Group 3 (n= 4) Group 4 (n= 44)
Group 5 (n= 3) Group 6 (n= 24)
(b)
Up-regulated genes in atxr5/6 at ZT4 and ZT16 (n= 164)
diurnal oscillation
no diurnal oscillation
65 99
120 0 120
Number of up-regulated genes
(c)
Group 1
WT ZT4 WT ZT16 atxr5/6 ZT4 atxr5/6 ZT16
(d)
Color Key
Group 2
WT ZT16 WT ZT4 atxr5/6 ZT4 atxr5/6 ZT16
(e)
Group 1
GO Term
double−strand break repair
DNA recombination
DNA damage response
nucleobase−containing compound metabolic process
genes 2.5 5.0 7.5 10.0 12.5
adjusted p−value 0.03 0.02 0.01
(f)
Group 2
GO Term
regulation of biological process
biological regulation
regulation of cellular process
regulation of proteasomal protein catabolic process
genes 2.5 5.0 7.5 10.0
adjusted p−value 0.025 0.020 0.015 0.010
(g)
Group 1
Up-regulated in atxr5/6 at ZT4 and ZT16
Diurnally oscillating
H3K27me1 at ZT4
H3K27me1 at ZT16
log2(H3K27me1/H3)
WT atxr5/6
-2.0 TSS TTS 2.0 Kb
(h)
Group 2
Up-regulated in atxr5/6 at ZT4 and ZT16
Non-diurnally oscillating
H3K27me1 at ZT4
H3K27me1 at ZT16
log2(H3K27me1/H3)
WT atxr5/6
-2.0 TSS TTS 2.0 Kb

Figure 3

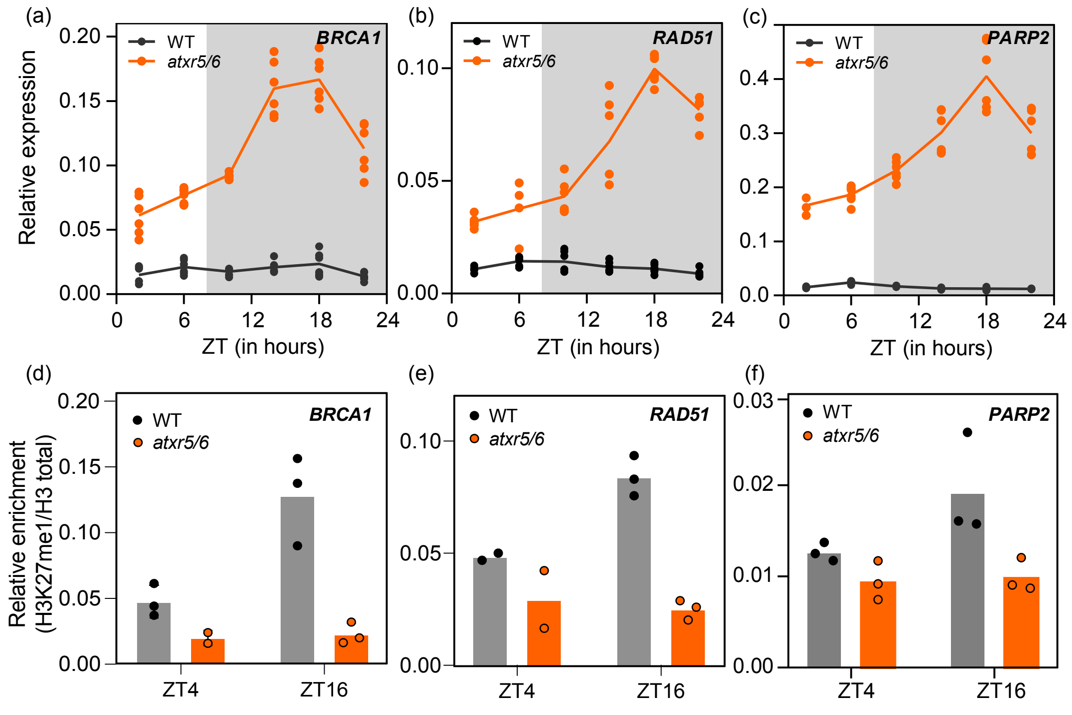

(a)
(b)
(c)
(d)
(e)
(f)
WT
atxr5/6
BRCA1
RAD51
PARP2
Relative expression
ZT (in hours)
Relative enrichment (H3K27me1/H3 total)
ZT4
ZT16

Figure 4

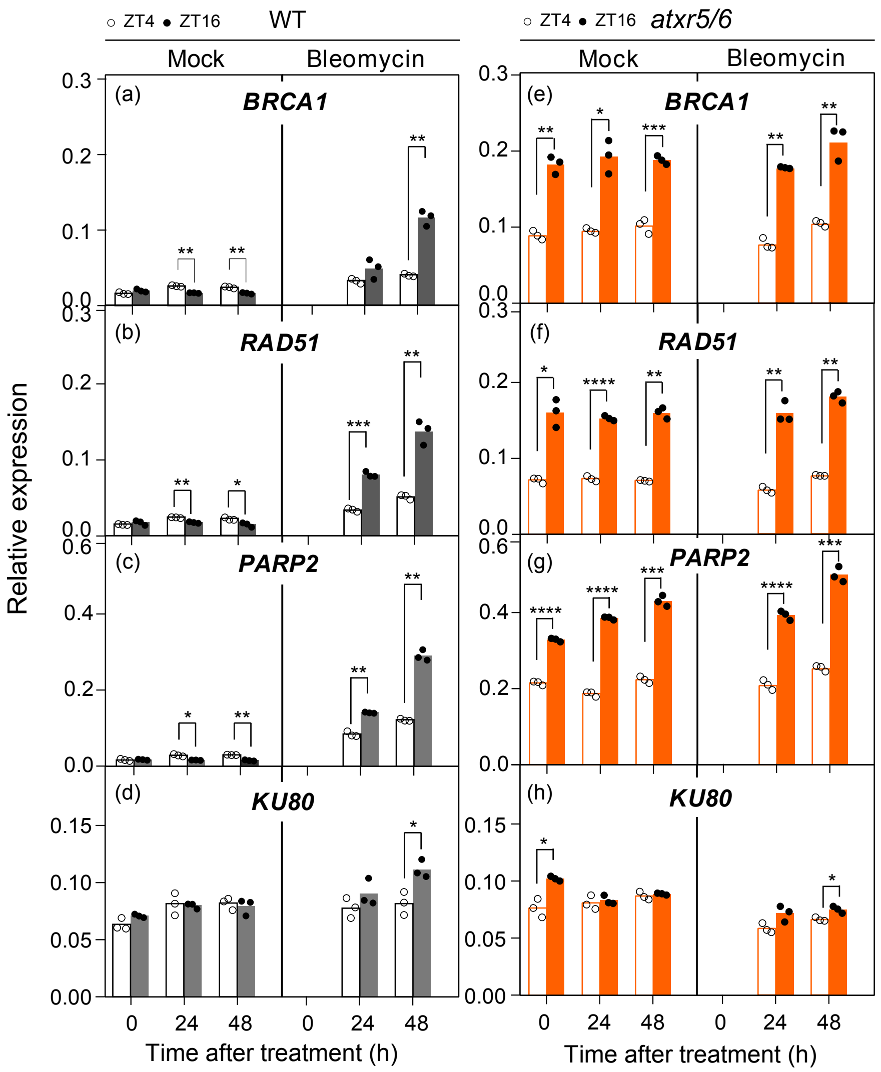
○ ZT4 ● ZT16 WT
○ ZT4 ● ZT16 atxr5/6
Mock
Bleomycin
(a) BRCA1
(b) RAD51
(c) PARP2
(d) KU80
(e) BRCA1
(f) RAD51
(g) PARP2
(h) KU80
Relative expression
0 24 48 0 24 48
Time after treatment (h)

Figure 5

Supplementary Information

Diurnal control of H3K27me1 deposition shapes expression of a subset of cell cycle and DNA damage response genes

Jorge Fung-Uceda+, María Sol Gómez+, Laura Rodríguez-Casillas, Anna González-Gil, Crisanto Gutierrez*

Centro de Biologia Molecular Severo Ochoa, CSIC-UAM, Nicolas Cabrera 1, Cantoblanco, 28049 Madrid, Spain

+ These are co-first authors

* Corresponding autor: Crisanto Gutierrez
Email: cgutierrez@cbm.csic.es
Telephone: +34 911964638

Keywords: H3K27me1, ATXR5/6, chromatin, histone modification, gene expression, diurnal cycles, DNA repair, euchromatin, Arabidopsis

This pdf file includes:
Figures S1-S9
Legends to Tables S1-3

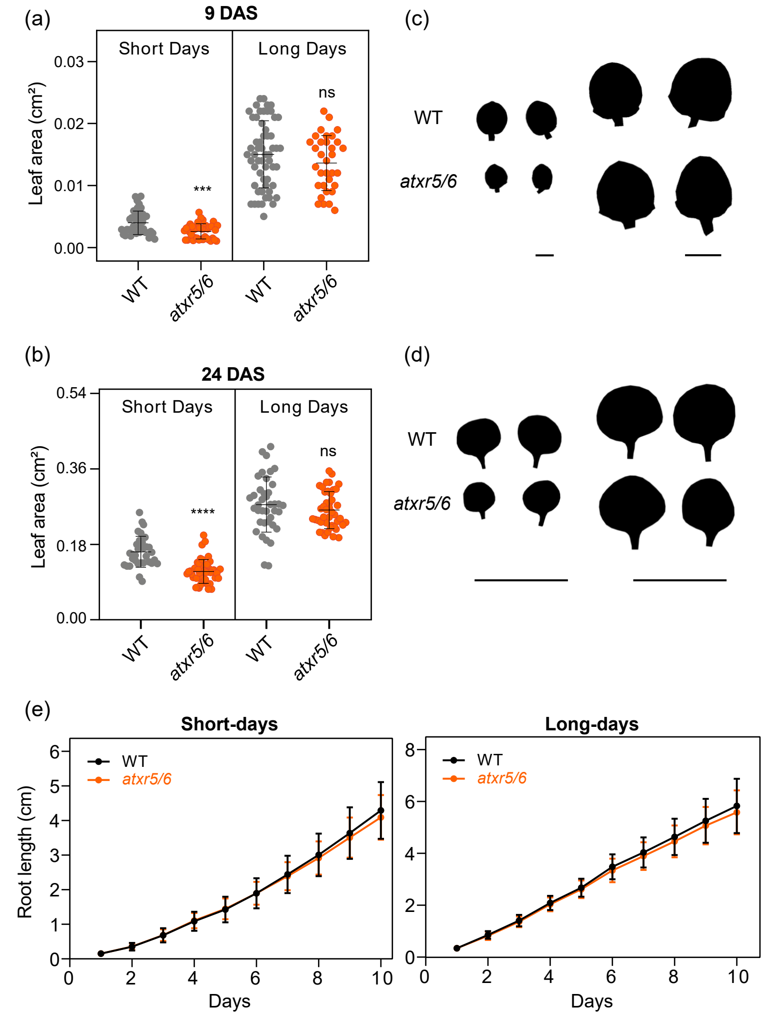

Figure S1. ***atxr5/6* mutants display growth phenotype only under specific photoperiodic conditions.** Leaf area in wild type (WT) and *atxr5/6* measured at (a) 9 DAS and (b) 24 DAS. Mean ± s.d. is shown (n≥ 33), **** p≤0.0001, *** p ≤0.001, ns p> 0.05. Representative images of WT and *atxr5/6* leaves at (c) 9 DAS and (d) 24 DAS grown under (left pictures in c-d) short-days and (right pictures in c-d) long-days. Black bars indicate the scale: (c) = 0.1cm and (d) = 1cm. (e) Root growth of WT and *atxr5/6* plants was scored over 10 days. In each case, mean ± s.d. is shown (short-days, WT, n = 89 roots; short-days, *atxr5/6*, n = 84 roots; long-days, WT, n = 46 roots; long-days, *atxr5/6*, n = 35 roots). Statistical analysis was carried out using a t-Student test. The results of two biological replicates are shown.

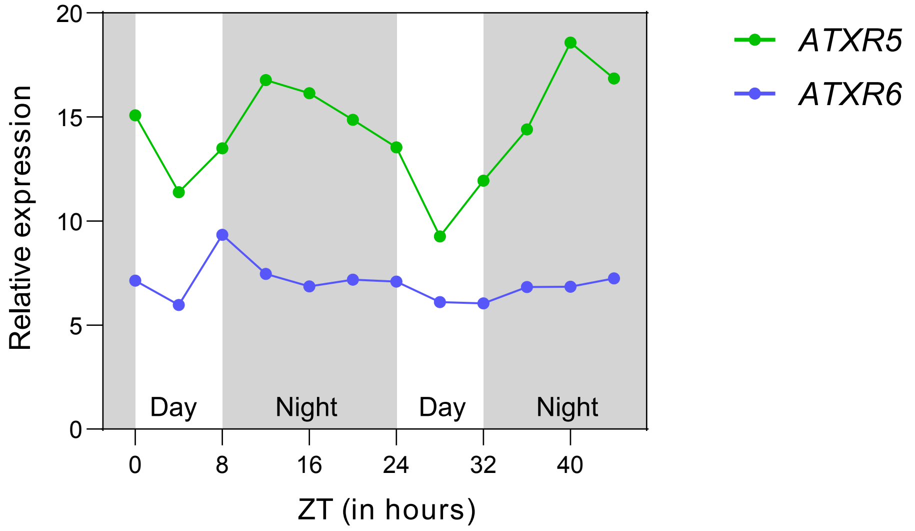

Figure S2. Diurnal expression of *ATXR5* and *ATXR6* genes under short-day growth conditions. Data were obtained from the diurnal project database (http://diurnal.cgrb.oregonstate.edu/).

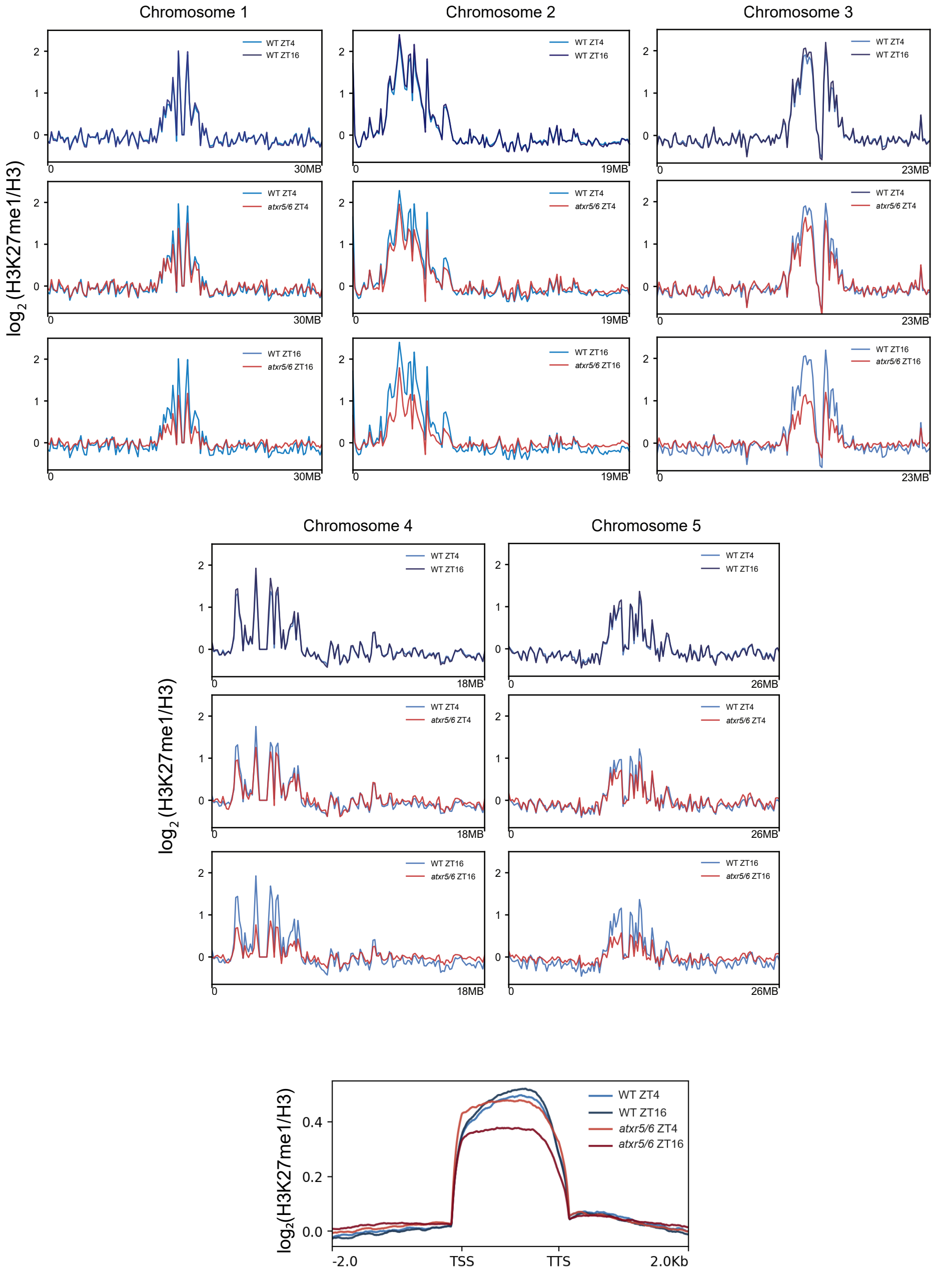

Figure S3. H3K27me1 profile along the Arabidopsis chromosomes and gene bodies.
Metaplots showing the H3K27me1 signal obtained in the ChIP-Seq experiments across the five Arabidopsis chromosomes (upper and middle panels) and across the gene bodies (bottom panel). Data shown in the figure were obtained from analysis using two merged independent biological replicates.

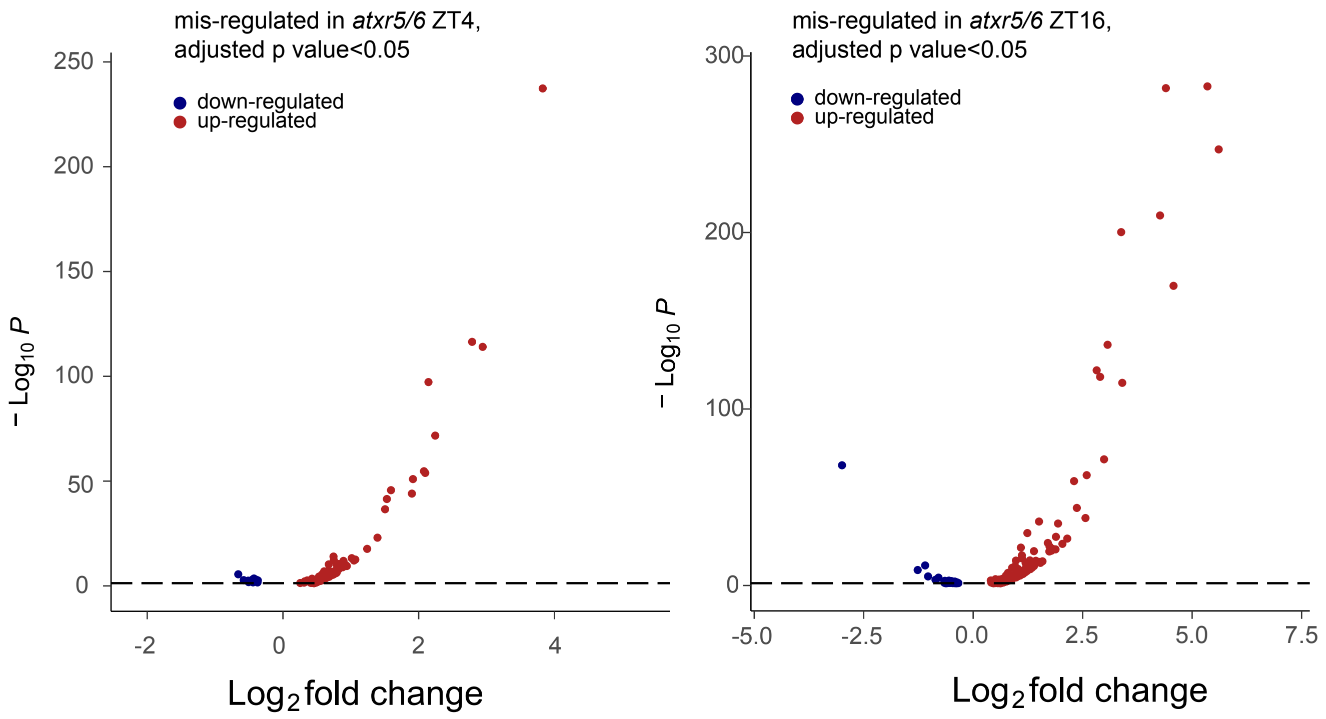

Figure S4. Volcano plots of RNA-Seq experiments.
Mis-regulated genes in the *atxr5/6* mutant were compared with the wild type (WT) from samples obtained at ZT4 (left panel) and ZT16 (right panel). The log2 fold-change indicates the mean expression level for each gene using an adjusted p value <0.05. Each dot represents one gene. Data shown in the figure were obtained from analysis using three merged independent biological replicates.

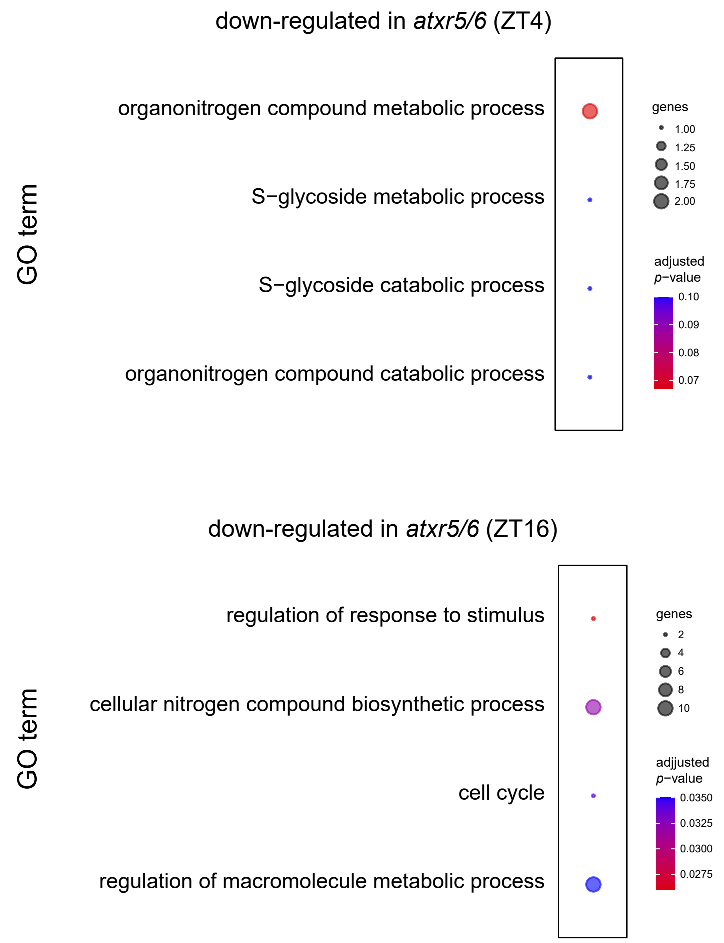

Figure S5. GO of down-regulated genes.
Gene ontology (GO) term analysis for down-regulated genes in *atxr5/6* at ZT4 (upper panel; 11 genes) and ZT16 (lower panel; 43 genes). The categories with higher number of significant annotated genes are shown. Data shown in the figure were obtained from analysis using three merged independent biological replicates.

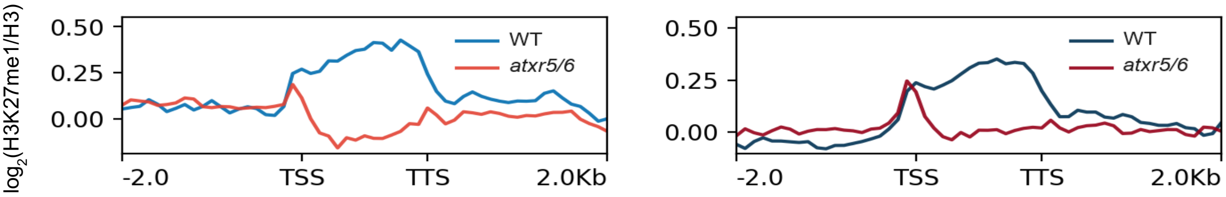

Figure S6. H3K27me1 profile of up-regulated genes in *atxr5/6* with detectable signal in wild type (WT) along the gene bodies. Metaplots showing the H3K27me1 signal obtained in ChIP-Seq experiments normalized to total H3 in WT and *atxr5/6* seedlings along the bodies of up-regulated genes with loss of H3K27me1 deposition in *atxr5/6*, at ZT4 (left) and ZT16 (right). 2kb of flanking upstream and downstream regions were included. Data shown in the figure were obtained from analysis using two merged independent biological replicates.

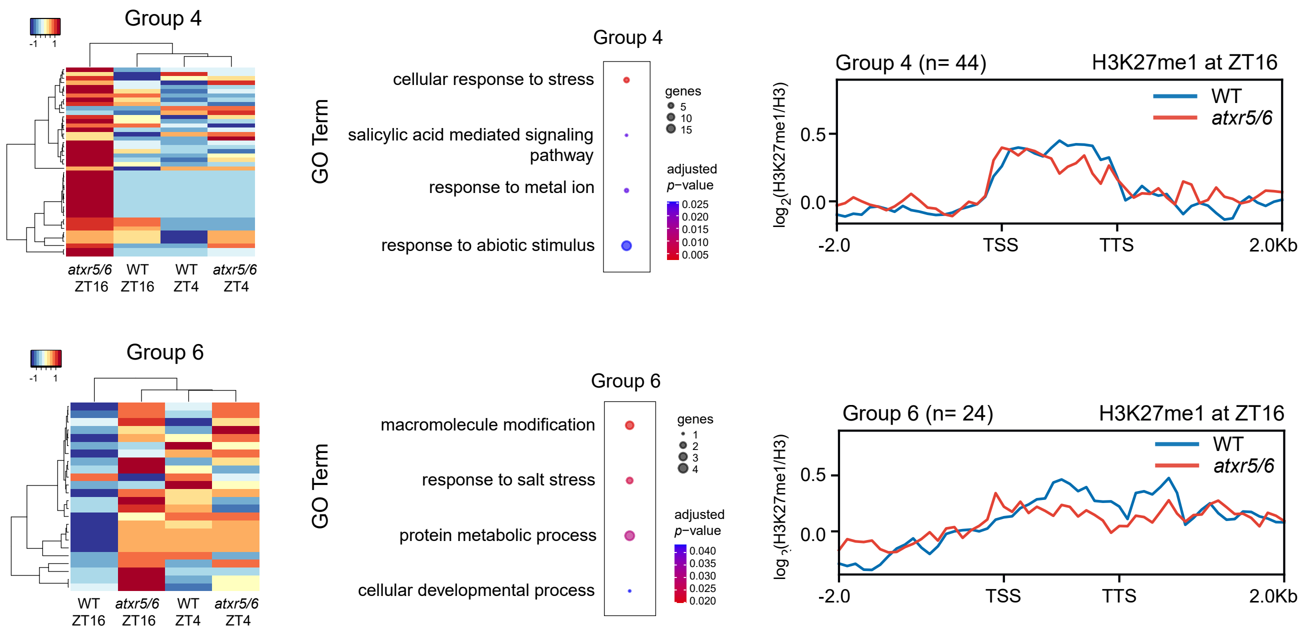

Figure S7. Heatmaps, gene ontology analysis and H3K27me1 profile of genes in groups 4 and 6, described in Fig. 3.

Clustered heatmap of gene expression level (left panels) in wild type (WT) and *atxr5/6* for groups 4 and 6. Significantly mis-regulated genes at ZT4 and ZT16 in *atxr5/6* compared to the WT were identified by RNA-Seq (adjusted *p*-value <0.05). Genes were clustered by row and column according to the mean TPM values of three biological replicates. Data were scaled following Euclidean distance. GO term analysis for up-regulated genes in *atx5/6* of groups 4 (44 genes) and 6 (24 genes). The 4 categories with higher number of significantly annotated genes are shown. Groups 3 and 5 have a very small number of genes and have been omitted. Profile of H3K27me1 ChIP-Seq signal normalized to total H3 (right panels) in WT and *atxr5/6* mutant along the bodies of *atxr5/6* upregulated genes in groups 4 and 6, including 2kb of flanking upstream and downstream regions. Reads of ChIP-Seq data were divided in bins of 25bp. Data shown correspond to two merged biological replicates.

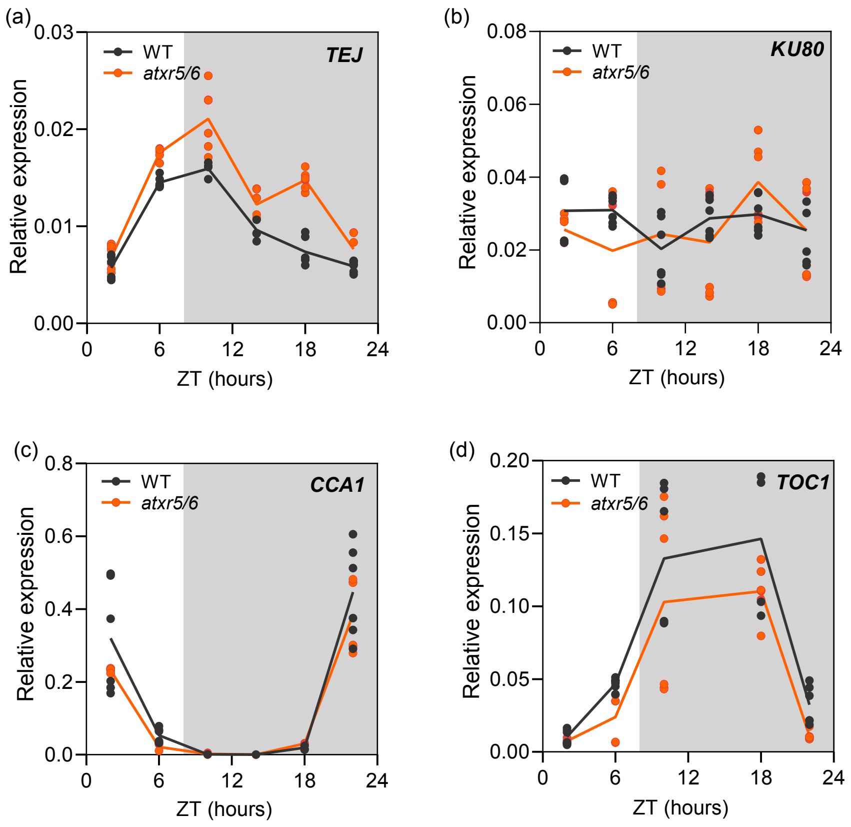

Figure S8. Diurnal time-course expression of *TEJ* (a), *KU80* (b), *CCA1* (c) and *TOC1* (d) in wild type (WT) and *atxr5/6* plants along the day.
Relative expression was obtained by real-time qPCR analyses. Individual data points from each biological replicate are shown together with the line joining the mean values. Data point shown come from two merged biological replicates.

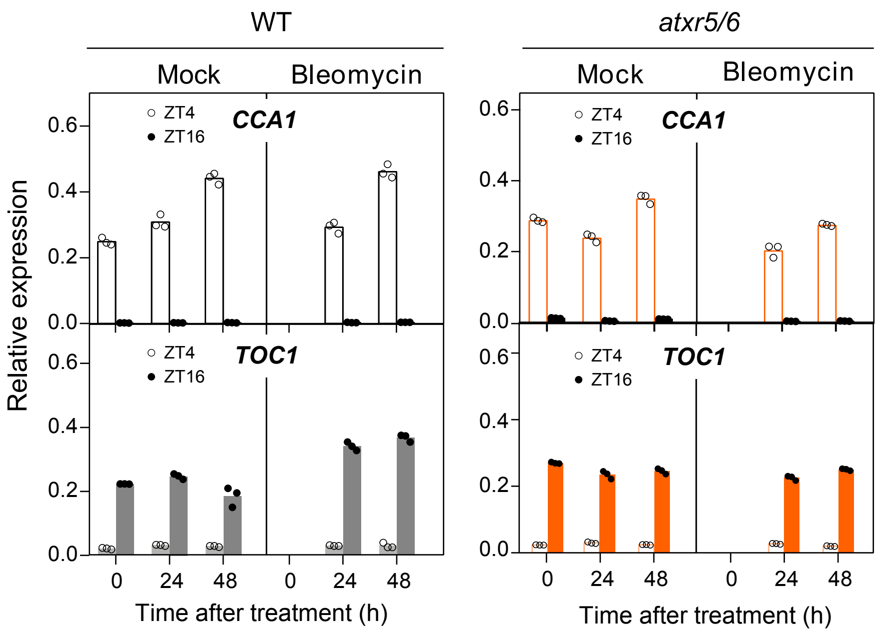

Figure S9. Diurnal oscillation of *CCA1* and *TOC1* genes after exposure to genotoxic agents. *CCA1* and *TOC1* expression in wild type (WT) and *atxr5/6* plants at ZT4 and ZT16 after bleomycin treatment. Plants were grown under short-day for 14 DAS. Relative expression was obtained by real-time qPCR analyses. Bars represent mean values of three technical replicates corresponding to one of the two biological replicates performed that rendered similar results.
